# Active surveillance versus treatment in low-risk DCIS: Women’s preferences in the LORD-trial

**DOI:** 10.1016/j.ejca.2023.113276

**Published:** 2023-10

**Authors:** Renée S.J.M. Schmitz, Ellen G. Engelhardt, Miranda A. Gerritsma, Carine M.T. Sondermeijer, Ellen Verschuur, Julia Houtzager, Rosalie Griffioen, Valesca Retèl, Nina Bijker, Ritse M. Mann, Frederieke van Duijnhoven, Jelle Wesseling, Eveline M.A. Bleiker, Grand Challenge PRECISION Consortium

**Affiliations:** a Division of Molecular Pathology, Netherlands Cancer Institute, Amsterdam, the Netherlands; b Division of Psychosocial Research and Epidemiology, Netherlands Cancer Institute, Amsterdam, the Netherlands; c Department of Biometrics, Netherlands Cancer Institute, Amsterdam, the Netherlands; d Dutch Breast Cancer Society (‘Borstkanker Vereniging Nederland’), Utrecht, the Netherlands; e Department of Radiation Oncology, Amsterdam University Medical Center, Amsterdam, the Netherlands; f Department of Radiology, Netherlands Cancer Institute – Antoni van Leeuwenhoek Hospital, Amsterdam, the Netherlands; g Department of Radiology, Radboud University Medical Center, Nijmegen, the Netherlands; h Department of Surgery, Netherlands Cancer Institute – Antoni van Leeuwenhoek Hospital, Amsterdam, the Netherlands; i Department of Pathology, Netherlands Cancer Institute – Antoni van Leeuwenhoek Hospital, Amsterdam, the Netherlands; j Department of Pathology, Leiden University Medical Center, Leiden, Netherlands; k Family Cancer Clinic, Netherlands Cancer Institute – Antoni van Leeuwenhoek Hospital, Amsterdam, the Netherlands; l Department of Clinical Genetics, Leiden University Medical Center, Leiden, Netherlands

**Keywords:** DCIS, Shared decision making, Patient preference

## Abstract

**Background:**

Ductal carcinoma in situ (DCIS) can progress to invasive breast cancer (IBC), but most DCIS lesions remain indolent. However, guidelines recommend surgery, often supplemented by radiotherapy. This implies overtreatment of indolent DCIS. The non-randomised patient preference LORD-trial tests whether active surveillance (AS) for low-risk DCIS is safe, by giving women with low-risk DCIS a choice between AS and conventional treatment (CT). Here, we aim to describe how participants are distributed among both trial arms, identify their motives for their preference, and assess factors associated with their choice.

**Methods:**

Data were extracted from baseline questionnaires. Descriptive statistics were used to assess the distribution and characteristics of participants; thematic analyses to extract self-reported reasons for the choice of trial arm, and multivariable logistic regression analyses to investigate associations between patient characteristics and chosen trial arm.

**Results:**

Of 377 women included, 76% chose AS and 24% CT. Most frequently cited reasons for AS were “treatment is not (yet) necessary” (59%) and trust in the AS-plan (39%). Reasons for CT were cancer worry (51%) and perceived certainty (29%). Women opting for AS more often had lower educational levels (OR 0.45; 95% confidence interval [CI], 0.22–0.93) and more often reported experiencing shared decision making (OR 2.71; 95% CI, 1.37–5.37) than women choosing CT.

**Conclusion:**

The LORD-trial is the first to offer women with low-risk DCIS a choice between CT and AS. Most women opted for AS and reported high levels of trust in the safety of AS. Their preferences highlight the necessity to establish the safety of AS for low-risk DCIS.

## Highlights

• The LORD-trial investigates whether active surveillance is safe in low-risk DCIS.
• Women strongly preferred active surveillance (76%) to conventional treatment (24%).
• Most cited reason for active surveillance was “treatment is not (yet) necessary”.
• Most cited reason for conventional treatment was "avoiding cancer worry".

## 1 Introduction

Ductal carcinoma in situ (DCIS) is a potential precursor to invasive breast cancer (IBC) [1], [2]. Its incidence has increased with the introduction of population-based breast cancer screening [3] and digital mammography [4], [5]. Currently, it constitutes 20% of all newly diagnosed breast neoplasms [6]. As DCIS has the potential to progress to IBC, it is treated like early-stage IBC. Current treatment guidelines advise surgery, either a mastectomy (MST) or breast-conserving surgery (BCS), often followed by radiotherapy (RT) and, in some countries, endocrine treatment [7]. However, up to 80% of DCIS lesions are indolent, low-risk lesions that will never progress to IBC during the patient’s lifetime [1], [8], [9], [10]. Consequently, there is a growing concern about overtreatment in women with DCIS [2], [9], [11], [12].

Previously reported factors for low-risk DCIS are the absence of symptoms, screen-detected presentation, higher age at diagnosis, and pathological low or intermediate grade [13], [14], [15]. The PRECISION CRUK Cancer Grand Challenge Consortium aims to reduce overtreatment for DCIS by refining the criteria to better distinguish low- from high-risk DCIS [16]. The LOw Risk DCIS (LORD)-trial was initiated in 2015 as a randomised controlled trial (RCT) studying non-inferiority of active surveillance (AS) compared to conventional treatment (CT) (Clinical trial number NCT02492607) [17], [18]. As recruitment was initially slow, in 2020, the LORD-trial was converted into a non-randomised patient preference trial. The primary endpoint remained the percentage of women without an occurrence of ipsilateral IBC after 10 years of follow-up in the AS arm compared to the CT arm.

The LORD-trial is the first study to offer women with low-risk DCIS a choice between either CT or AS, thus creating a unique opportunity to gain insight into factors associated with women’s preference for either AS or CT.

As there currently are no well-validated prediction models providing valid and reliable, accurate prognostic estimates available for women with low-risk DCIS [19], perception of the risk of progression to IBC varies widely, both among patients and their clinicians [20]. Moreover, in a discrete choice experiment, not only a difference in risk perception was shown between patients and clinicians, but also in the weight given to the risk of progression in decision making [20]. For clinicians, risk of IBC was the major determinant for preference of either arm, whereas for patients the risk of IBC was the least important factor [20]. The results of this study raised questions about factors impacting women’s preference for CT vs. AS.

To answer these questions, in this article, we a) analysed the preferred trial arm for each LORD-trial participant, b) identified their motives for opting for either trial arm, and c) assessed whether patient and disease characteristics were associated with said preference.

## 2 Methods

### 2.1 Study population

The current study is embedded within the ongoing LORD patient preference trial for which women are being recruited in 52 hospitals across the Netherlands. Briefly, women of 45 years or over, with an American society of Anesthesiologists classification score (ASA) 1–2 and with unilateral, pure DCIS, grade one or two, any size, detected through screening, appearing on mammography as calcifications only, Estrogen receptor (ER)-positive Human epidermal growth factor 2 (HER2)-negative, can be included. Excluded are women with symptomatic DCIS, a history of (breast) malignancy or DCIS, and women (or family members) with a proven mutation increasing the risk of breast cancer. Women eligible for the LORD study who had completed the baseline questionnaire by 17th June 2022 and had made a choice between trial arms at questionnaire completion were selected for the current study.

CT in the LORD-trial follows Dutch treatment guidelines and consists of surgery, either MST or BCS. RT can be prescribed after BCS at the discretion of the treating clinician as per local policy. No restrictions on target volume, dose, and fractionation apply.

The LORD-trial was reviewed by the medical research ethics committee of the Netherlands Cancer Institute (NL55612.031.16).

### 2.2 Procedures and measures

Information regarding patients’ characteristics (i.e. age, educational level, employment status, smoking habits, trust in oncologist, perceived level of shared decision making (SDM), tolerance of uncertainty (TOU), level of anxiety, perception of the risk of developing IBC) were collected with the baseline study questionnaire as well as their trial arm preference and motivation for preference. Patients received the baseline questionnaire immediately after consultation with their breast surgeon and/or nurse practitioner in which the diagnosis and DCIS management strategies were discussed. Details on the questions and answer categories in the patient questionnaire are described in the supplementary methods.

Educational level [21] was categorised in three levels: low level (i.e. elementary school, secondary vocational education), moderate level (i.e. high school, post-secondary vocational education) and high level (i.e. higher vocational education or university). Employment status was summarised into: unemployed; working; retired. Relationship status was summarised in two categories: single or in a relationship. Smoking habits were categorised as never, currently a smoker, and not anymore. Perceived level of SDM was categorised as final decision made by: patient, oncologist, together, and other. Trust in oncologist was measured using the abbreviated, five-item “Trust in Oncologist Scale” by Hillen et al. [22], [23], providing a final rating categorised in little trust, neutral and much trust. TOU was measured using the Intolerance of Uncertainty Scale [24], [25]. For these analyses, we categorised high versus low intolerance of uncertainty at the cut off of 75% of the maximum achievable score. Perception of the risk of developing IBC was summarised in three categories for analyses: lower; equal; higher than the average Dutch woman. Level of anxiety was measured using the Hospital Anxiety and Depression Scale (HADS) [26]. Scores were summarised in two categories: not elevated and elevated, in which a score above 10 was defined as an elevated score.

Clinical data were collected by trained data managers from patients’ electronic health records. For this study, DCIS-grade and DCIS-size were extracted from the LORD-trial’s electronic data capture system. DCIS-grade was defined as grade one or two following the WHO classification of breast tumours [27]. DCIS-size was defined as the size of the largest diameter of calcifications on mammography.

### 2.3 Statistics

Statistics Descriptive statistics were used to describe the participant characteristics. Fisher’s exact tests were used to test potential differences in distribution of patient characteristics between DCIS trial arms (i.e. AS and CT). A qualitative thematic analysis was performed to extract the underlying themes in the reasons participants reported for selecting either trial arm. Labels were double-coded by two researchers (RSJMS, EGE) independently and discrepancies were resolved through consensus. Up to three reasons for treatment preference were identified per patient.

Multivariable logistic regression analyses were performed to identify factors associated with choice for AS. Factors considered were: age at diagnosis, DCIS-grade, DCIS-size, educational level, trust in oncologist, perceived level of SDM, TOU, HADS score for anxiety, and perception of risk of developing IBC. P-value ≤ 0.05 was considered statistically significant. All analyses were performed using STATA/SE 15.0 (StataCorp LP, College Station, TX).

## 3 Results

### 3.1 Treatment distribution

Questionnaires were available for 384 women, from which 377 (98%) reported their choice regarding trial arms. There were no notable differences between women who had reported their trial arm preference (n = 377) and those who had not (n = 7). Patient characteristics are reported in Table 1. Median age at diagnosis was 59 years, 50% of DCIS lesions were small (<20 mm), 34% had grade I DCIS, 39% grade II and for 28% DCIS-grade was either grade I or II, but not yet recorded in the trial’s electronic data capture system. Educational level was low in 35%, intermediate in 30%, and high in 35% of women, which differed significantly between trial arms (p = 0.015). Out of 377 women included, 288 (76%) reported AS as their preferred trial arm, whereas 89 (24%) opted for CT.

**Table 1:** Patient and ductal carcinoma in situ (DCIS) characteristics.

	All patients n = 377	Active surveillance n = 288	Conventional treatment n = 89	
Age (median)	59	59	57	
	n (%)	n (%)	n (%)	p-value^a^

Age				0.188
45–54 55–64 65–74 75–84	134 (36) 125 (33) 109 (29) 9 (2)	99 (34) 92 (32) 88 (31) 9 (3)	35 (39) 33 (37) 21 (24) 0	
DCIS grade				0.184
Grade 1 Grade 2 Not yet registered	126 (34) 146 (39) 105 (28)	93 (32) 108 (38) 87 (30)	33 (37) 38 (43) 18 (20)	
DCIS size				0.532
< 20 mm 20–49 mm ≥ 50 mm Not yet registered	190 (50) 45 (12) 13 (3) 129 (34)	142 (49) 32 (11) 11 (4) 103 (36)	48 (54) 13 (15) 2 (2) 26 (29)	
Educational level^b^				0.015
Low Intermediate High	130 (35) 114 (30) 133 (35)	110 (38) 85 (30) 93 (32)	20 (22) 29 (33) 40 (45)	
Employment status				0.495
Unemployed Working Retired	75 (20) 227 (60) 75 (20)	62 (22) 168 (58) 58 (20)	13 (15) 59 (66) 17 (19)	
Relationship status				0.750
Single In a relationship	66 (18) 311 (83)	52 (18) 236 (82)	14 (16) 75 (84)	
Smoking				0.706
Never Currently a smoker Not anymore	174 (46) 47 (13) 155 (41)	131 (46) 35 (12) 122 (42)	43 (49) 12 (14) 33 (38)	
Tolerance of uncertainty				0.680
High tolerance Low Tolerance	278 (74) 99 (26)	214 (74) 74 (26)	64 (72) 26 (28)	
HADS score for anxiety				0.135
Not elevated Elevated	317 (84) 60 (16)	247 (86) 41 (14)	70 (79) 19 (21)	

Abbreviation: mm, millimetre.

a p-values were calculated using Fisher’s exact tests.

b Educational level was categorised in three levels: low level (i.e. elementary school, secondary vocational education), moderate level (i.e. high school, post-secondary vocational education) and high level (i.e. higher vocational education or university).

### 3.2 Motives for trial arm preference

**Fig. 1:**
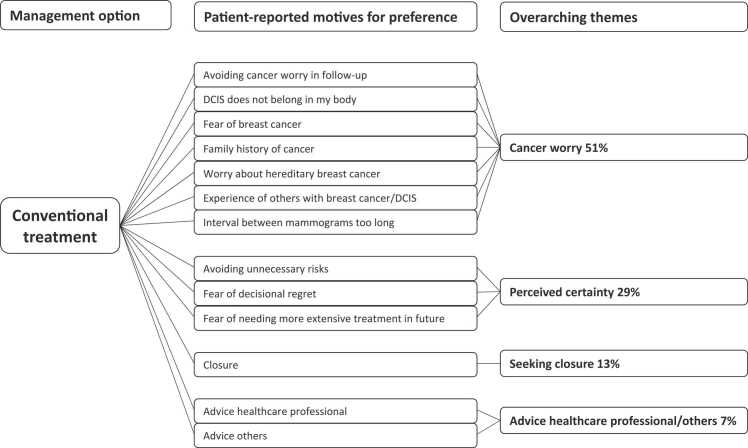
Patient-reported motives for preferring conventional treatment (n = 89 women).

**Fig. 2:**
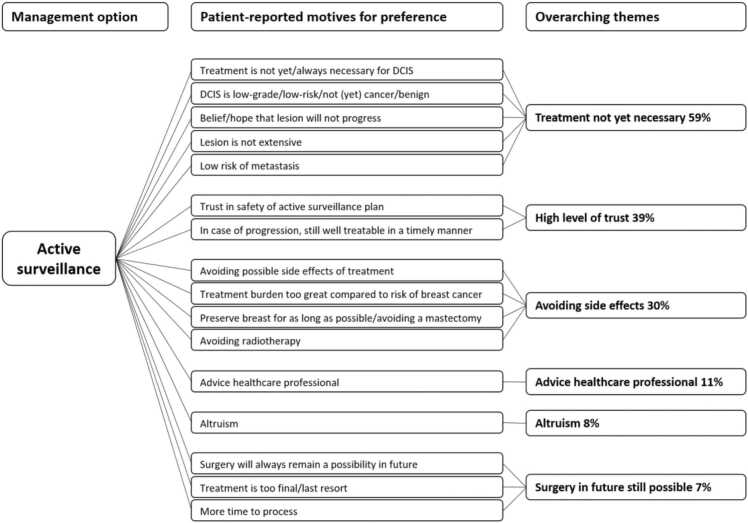
Patient-reported motives for preferring active surveillance (n = 288 women).

Motives for trial arm preference reported by women who opted for CT are reported in Fig. 1. In women opting for CT, the 51% reported themes related to cancer worry and 29% related to achieving certainty. The main motives reported by women who opted for AS were treatment for DCIS is not yet/always necessary (59%), trust in safety of the follow-up strategy (39%), and avoiding treatment side effects (30%) (Fig. 2). Eight percent of women reported altruism (e.g. “help women in the future”) as reason to choose AS.

### 3.3 Associations of patient and disease characteristics with preference of DCIS management strategy

In multivariable logistic regression (Table 2), women who opted for AS were less often highly educated (OR, 0.49; 95% confidence interval [CI], 0.24–1.00) compared to women opting for CT. Additionally, they more often reported that the final “treatment” decision was a shared decision between them and their oncologist (OR 2.55; 95% CI, 1.30–5.00) as opposed to their individual decision. Further, they were more often neutral (OR, 9.08; 95% CI, 1.61–51.22) in trusting in their physician, compared with women choosing CT. Age at diagnosis, DCIS-size, DCIS-grade, TOU, trust in healthcare provider, HADS score for anxiety, and perception of breast cancer risk were not statistically significantly associated with preference for either management option in the multivariate analyses (Table 2).

**Table 2:** Association between patient and disease characteristics and preference for active surveillance, univariate and multivariable logistic regression analyses.

Variable	n women	Univariate OR (95% CI)	p-value	Multivariable OR (95% CI)	p-value
Age at diagnosis		1.02 (1.00–1.06)	0.102	1.01 (0.97–1.04)	0.683
DCIS-grade					
Grade 1	126	1.00 (ref)		1.00 (ref)	
Grade 2	146	1.01 (0.59–1.74)	0.976	1.07 (0.60–1.91)	0.818
Unknown Grade^a^	105	1.72 (0.90–3.27)	0.101	2.25 (0.76–6.65)	0.141
DCIS-size					
< 20 mm	190	1.00 (ref)		1.00 (ref)	
20–49 mm	45	0.83 (0.40–1.71)	0.618	0.88 (0.40–1.94)	0.744
> 49 mm	13	1.86 (0.40–8.69)	0.431	2.19 (0.38–12.63)	0.380
Unknown Size^a^	129	1.34 (0.78–2.30)	0.290	0.80 (0.30–2.15)	0.662
Educational level^b^					
Low	130	1.00 (ref)		1.00 (ref)	
Moderate	114	0.53 (0.28–1.01)	0.052	0.64 (0.31–1.33)	0.232
High	133	0.42 (0.23–0.77)	0.005	0.45 (0.22–0.93)	0.030
Trust in oncologist					
Little trust	21	1.00 (ref)		1.00 (ref)	
Much trust	327	2.95 (1.21–7.21)	0.018	2.16 (0.81–5.75)	0.124
Neutral	29	12.27 (2.31–63.34)	0.003	9.08 (1.61–51.22)	0.012
Decision made by					
Patient	244	1.00 (ref)		1.00 (ref)	
Oncologist	5	1.51 (0.17–13.80)	0.713	0.95 (0.08–10.7)	0.967
Together	99	2.50 (1.31–4.79)	0.005	2.71 (1.37–5.37)	0.004
Other^c^	29	0.99 (0.42–2.35)	0.954	0.87 (0.34–2.18)	0.759
Tolerance of uncertainty					
High tolerance	278	1.00 (ref)		1.00 (ref)	
Low Tolerance	99	0.89 (0.52–1.51)	0.654	0.87 (0.47–1.60)	0.651
HADS anxiety score					
Not elevated	317	1.00 (ref)		1.00 (ref)	
Elevated	60	0.61 (0.33–1.12)	0.111	0.60 (0.29–1.24)	0.168
Risk perception^d^					
Equal	146	1.00 (ref)		1.00 (ref)	
Lower	11	3.40 (0.42–27.42)	0.252	4.71 (0.52–42.4)	0.167
Higher	206	1.21 (0.74–2.00)	0.445	1.42 (0.83–2.44)	0.206
Don’t know	14	0.45 (0.15–1.39)	0.166	0.55 (0.15–1.97)	0.360

Abbreviations: n, number; OR, Odds Ratio; CI, Confidence Interval; DCIS, Ductal Carcinoma In Situ; HADS, Hospital Anxiety and Depression Scale.

a Not yet registered in trial database.

b Educational level was categorised in three levels: low level (i.e. elementary school, secondary vocational education), moderate level (i.e. high school, post-secondary vocational education) and high level (i.e. higher vocational education or university).

c Other was accompanied by open-ended question, most reported including family and friends in the decision-making process.

d Perception of breast cancer risk compared to other women of the same age who did not have DCIS.

## 4 Discussion

The LORD-trial is the first trial to offer women with low-risk DCIS the choice between AS CT, thus providing unique data on preferences of women with low-risk DCIS, revealing a strong preference for AS (76%) compared to CT (24%). Reasons reported by participants for choosing AS revolved around participants’ belief that treatment was not (yet) necessary and a high level of trust in the safety of the AS strategy. Women who chose CT seemed to be driven by a wish to avoid cancer worry in and perceived certainty. Women opting for AS were less often highly educated and more often experienced SDM compared to women opting for CT.

Our study has a number of strengths. Firstly, the questionnaires included both well-established items, such as the HADS anxiety score [26] and intolerance of uncertainty [24], as well as open-ended questions in order to fully capture the patient’s reasoning behind their preferences. Secondly, all written motives for trial arm selection were double coded by two researchers separately and discrepancies were resolved through consensus. Thirdly, because women were offered the questionnaires directly after the consultation with their treating physician, their answers had not yet been influenced by experiencing potential (side) effects of treatment. Lastly and most importantly, while patients of lower educational levels are often underrepresented in trials, especially those including questionnaires, women of all educational levels were equally represented in our study.

As the LORD-trial is the first patient preference trial offering AS and CT, studies to compare our results with are lacking. However, focus groups in preparation for the randomised DCIS de-escalation trial LORIS [28], showed about a third of women approached for the hypothetical trial would prefer AS over CT [29]. Furthermore, for other lesions where AS is offered, such as cervical intraepithelial neoplasia (CIN) [30], [31] and low-risk prostate cancer [32], [33], several studies have been reported. In an RCT offering 1638 “healthy” participants hypothetical scenarios on management of CIN, 79% opted for AS. Most reported reasons for AS were potential negative side effects of surgery and trust in their clinician, which is in line with our findings [34]. Similar to our study, several studies in the context of prostate cancer report that men who had a preference for AS experienced more SDM compared to men opting for CT [35], [36] and reported higher trust in their clinician [37]. Furthermore, although potential side effects of treatment are considerably different, men with low-grade prostate cancer also reported avoiding side effects as an important reason for preferring AS over active treatment [36]. Notably, an important motive in men opting for active treatment is cancer eradication/preventing cancer worry [38] which is also in line with the findings in our study.

The strong preference for AS in women participating in the LORD-trial has been previously reported in a discrete choice experiment among LORD-trial participants [20]. In contrast, in a Hong Kong-based prospective cohort study, women with various breast diagnoses were interviewed about the option of AS if proven safe. In this study, most women (89%) would opt for CT, mostly due to anxiety [39]. These contradictory findings could be due to cultural differences or might be explained by the inclusion of women who had been previously diagnosed with a breast lesion. Of these, women with a history of breast cancer were more likely to prefer CT compared to women previously diagnosed with DCIS (p = 0.0034).

In general, perceived patient autonomy was high in both groups, as 65% of all women reported making the final decision regarding their DCIS management themselves and 26% reported they made their decision together with their oncologist. Only 1.3% of women indicated that their oncologist made the final decision. While women opting for AS more often experienced SDM, women choosing CT more often reported making the final decision themselves. However, an important consideration in studies regarding patient preference and SDM is that it is often difficult to disentangle patients’ preferences from the (perceived) recommendation from their treating physician. Especially considering women opting for AS were more often lower educated, which has previously been reported to be associated with more trust in their physician [40], suggesting women with a lower education might be more inclined to follow the physicians (perceived) preference. Existing literature has shown that physicians (often subconsciously) tend to steer patients into a direction which they feel is in their best interest [41], [42]. As consultations are typically not observed or recorded, the extent of (subconscious) steering by physicians remains unclear as is the extent to which SDM is appropriately applied. Although women in the trial all receive educational flyer, written in accessible language by the patient representatives of the trial, a well-designed patient decision aid would be a helpful addition in improving informed decision making. However, no patient decision aids including the option of AS are available for Dutch patients [19]. It is therefore difficult to assess to what extent the perceived high level of SDM would be in line with objective measures for SDM in these patients.

A limitation of our study was that women with low-risk DCIS who were not asked to participate in the LORD-trial, decided not to participate, or were diagnosed in a non-participating site, did not receive the questionnaires. Therefore, we cannot fully determine to what extent the results may be applied to all women with low-risk DCIS. However, as the trial also includes the option of CT, women not in favour of the experimental arm can still participate, limiting the risk of potential bias introduced by including only women in favour of the experimental arm. Moreover, as 69% of all hospitals providing breast cancer care in the Netherlands currently take part in the LORD-trial, the majority of women with low-risk DCIS in the Netherlands are recruited in the trial. Another limitation might be that the LORD-trial is a national study, meaning these results might not necessarily be applicable to other cultures and countries as treatment preferences and attitude towards de-escalation might differ. However, the increasing attention for de-escalation of DCIS-management in literature [2], [43], [44], [45], [46], indicates that this interest extends beyond the Netherlands.

The LORD-trial is still recruiting patients at a high pace and even though the final outcome analysis will not be done until ten years of follow-up are completed, the current distribution of 76% in favour of AS means many women are already omitting CT. Currently, high-quality decision aids for women with (low-risk) DCIS, including the option of AS, are lacking [19]. As such, development of new decision aids to aid in SDM is vital to ensure patients make a well-informed choice, reducing their risk for potential decisional regret and its negative impact on their quality of life (QoL).

## 5 Conclusions

While results regarding primary outcomes of the LORD-trial are pending, these patient-reported data already provide unique insights in real time on patients’ treatment preferences and, in future studies, their potential impact on their overall health, healthcare use and quality of life. The evident patient preference for AS in the LORD-trial highlights the need for evidence on whether AS for low-risk DCIS is indeed safe, and thus the importance of DCIS treatment de-escalation trials. Anticipating on the trial results, novel decision aids should be developed to aid patients and clinicians to make informed decisions about the management of low-risk DCIS.

## Funding

This work was supported by the Dutch Cancer Society, KWF kankerbestrijding [grant numbers NKI 2014-7167; KWF NKI 2021-15093] and The 10.13039/100013421Pink Ribbon Foundation [grant number: 2014-183 WO 54 PR130027]. Research at the Netherlands Cancer Institute is supported by institutional grants of the Dutch Cancer Society and of the 10.13039/501100002999Dutch Ministry of Health, Welfare and Sport.

## Ethics statement

The LORD-trial was reviewed by the medical research ethics committee of the Netherlands Cancer Institute (NL55612.031.16).

## CRediT authorship contribution statement

**R.S.J.M Schmitz:** Conceptualization, Methodology, Formal analysis, Data collection and curation, Writing – original draft, Visualization, Project administration. **E.G. Engelhardt:** Conceptualization, Methodology, Formal analysis, Data collection and curation, Writing – original draft, Visualization. **M.A. Gerritsma:** Data collection and curation, Writing – review & editing. **C.M.T. Sondermeijer:** Writing – review & editing. **E. Verschuur:** Writing – review & editing. **J. Houtzager:** Data collection and curation, Writing – review & editing. **R. Griffioen:** Data collection and curation, Writing – review & editing. **V. Retèl:** Writing – review & editing. **N. Bijker:** Writing – review & editing. **R.M. Mann:** Writing – review & editing. **F. van Duijnhoven:** Writing – review & editing. **J. Wesseling:** Conceptualization, Writing – review & editing, Supervision, Funding acquisition. **E.M.A. Bleiker:** Conceptualization, Writing – review & editing, Supervision.

## Declaration of Competing Interest

The authors declare that they have no known competing financial interests or personal relationships that could have appeared to influence the work reported in this paper.
