# Supplementary material for: Active surveillance versus treatment in low-risk DCIS: Women’s preferences in the LORD-trial
Source: Eur J Cancer. 2023 Oct;192:None. doi: 10.1016/j.ejca.2023.113276 (PMC10632767; doi:10.1016/j.ejca.2023.113276)
Supplement: Supplementary file 1 — Supplementary material [file mmc1.docx]

**Supplementary method section**

Information on patients’ preference for DCIS management strategy was collected using a multiple-choice question (i.e., “Which management option have you chosen?”) with the following answering categories: mastectomy; breast conserving surgery with radiotherapy; breast conserving surgery without radiotherapy; no surgery, active surveillance for 10 years; “decision is not yet definitive but I’m leaning toward…”. A self-developed open-ended question was used to assess patients’ motivation for their treatment preference (i.e., “I choose active surveillance/conventional treatment because...”). Educational level^1^ was measured using a widely used multiple-choice question (i.e., “What is your highest completed educational level?”) and categorized in three levels: low level (i.e., elementary school, secondary vocational education), moderate level (i.e., high school, post-secondary vocational education) and high level (i.e., higher vocational education or university). Employment status was measured using a self-developed multiple-choice question (i.e., “Do you currently have a job?”) including the answering categories: no; yes, fulltime; yes, part-time, that is … hours; no, I am retired. Answers were summarized into: unemployed; working; retired. Relationship status was measured using a self-developed multiple-choice question (i.e., “What is your marital status?”) including the following answering categories: single; widow; in a relationship; “other, that is …”. These were summarized in two categories: single or in a relationship. Smoking habits were measured using a self-developed multiple-choice question (i.e., “Do you currently smoke cigarettes?”) including the answering categories: no, I have never smoked; no, I quit smoking … years ago; yes, I smoke … cigarettes per week. Perceived level of shared decision making was measured using a multiple choice question based on the Control Preference Scale^2^ (i.e., “ Who made the decision on which treatment you will undergo for your DCIS” with the following answering categories: I decided; my oncologist decided; I decided together with my oncologist; “other, that is…”.Trust in oncologist was measured using the abbreviated, five-item “Trust in Oncologist Scale” by Hillen et al. ^3,4^, which consists of five items scored on a scale from completely disagree to completely agree. The score is calculated by adding the score for the five items together and dividing that by five. This provides a final rating between one and five, with higher scores reflecting greater trust. Tolerance of uncertainty (TOU) was measured using the Uncertainty Intolerance Scale (IUS)^5,6^. The IUS consists of 12 items, scored from completely disagree to completely agree. The total score is calculated by adding the scores for all the items together and the sum score ranges between 12 and 60. There is no official cut-off for the IUS, therefore, a data-driven cut off was used in this study. Here we defined high vs. low intolerance of uncertainty as follows: sum scores ≥ 36 (i.e., 75% of the maximum achievable score) were considered to reflect high intolerance for uncertainty and sum scores < 36 reflect low intolerance for uncertainty. Perception of the risk of developing IBC was measured using a frequently used multiple-choice question (i.e., “Compared with the average woman of your age from the Dutch population, you think your risk of developing breast cancer is...”) with the following answering categories lower; equal; slightly elevated; moderately elevated; highly elevated. These answers were summarized in three categories for analyses: lower; equal; higher. Level of anxiety was measured using the Hospital Anxiety and Depression Scale (HADS)^7^. The official cut offs state an anxiety score of seven or lower is considered “No clinical anxiety disorder”, eight to ten is considered “possible/mild clinical anxiety disorder” and >10 is considered “clinical anxiety disorder”^7^. For these analyses, scores were summarized in two categories: not elevated and elevated, in which a score above 10 was defined as an elevated HADS anxiety score.

**References**

1. Commission E. Eurydice National Education Systems. Updated 06-30-2023. Accessed 07-13, 2023. <https://eurydice.eacea.ec.europa.eu/national-education-systems/netherlands/overview>

2. Degner LF, Kristjanson LJ, Bowman D, et al. Information needs and decisional preferences in women with breast cancer. *JAMA: Journal of the American Medical Association*. 1997;277:1485-1492. doi:10.1001/jama.277.18.1485

3. Hillen MA, Koning CC, Wilmink JW, et al. Assessing cancer patients' trust in their oncologist: development and validation of the Trust in Oncologist Scale (TiOS). *Support Care Cancer*. Aug 2012;20(8):1787-95. doi:10.1007/s00520-011-1276-8

4. Hillen MA, Postma RM, Verdam MG, Smets EM. Development and validation of an abbreviated version of the Trust in Oncologist Scale-the Trust in Oncologist Scale-short form (TiOS-SF). *Support Care Cancer*. Mar 2017;25(3):855-861. doi:10.1007/s00520-016-3473-y

5. Carleton RN, Norton MA, Asmundson GJ. Fearing the unknown: a short version of the Intolerance of Uncertainty Scale. *J Anxiety Disord*. 2007;21(1):105-17. doi:10.1016/j.janxdis.2006.03.014

6. Helsen K, Van den Bussche E, Vlaeyen JW, Goubert L. Confirmatory factor analysis of the Dutch Intolerance of Uncertainty Scale: Comparison of the full and short version. *J Behav Ther Exp Psychiatry*. Mar 2013;44(1):21-9. doi:10.1016/j.jbtep.2012.07.004

7. Zigmond AS, Snaith RP. The hospital anxiety and depression scale. *Acta Psychiatr Scand*. Jun 1983;67(6):361-70. doi:10.1111/j.1600-0447.1983.tb09716.x
